# Subcutaneous Injections of Arsenious Acid in Skin Diseases

**Published:** 1871

**Authors:** 


Subcutaneous Injections of Arsenious Acid in Skin Diseases. Dr. Lipp publishes, in the *Arch. fur Dermat. und Syph.*, Nov. 3, 1869, two cases of psoriasis and three of chronic eczema, which were cured by hypodermic injections of arsenious acid. In the former the result of the injections was satisfactory, after the internal use of Fowler's solution had failed. The cases of eczema are not so conclusive, as other means besides the injections were used. In the first case of psoriasis, eight grains of arsenious acid were injected in forty-eight days, and in the second, four grains in thirty-eight days. The author gives minute details respecting the phenomena observed during the injections, and states that he does not mean to infer from so few cases the superiority of the injections over the internal use of arsenic; but he merely observes that in favor of the former he might mention—the certainty of absorption, the non-interference with the organs of digestion, the small doses used, and the short treatment. As quinine and other remedies are now frequently injected, a time will probably soon come when the stomach will rarely be troubled with medicinal substances.

